# Prelimbic cortical pyramidal neurons to ventral tegmental area projections promotes arousal from sevoflurane anesthesia

**DOI:** 10.1111/cns.14675

**Published:** 2024-03-15

**Authors:** Fuyang Cao, Yongxin Guo, Shuting Guo, Xinyu Hao, Lujia Yang, Jiangbei Cao, Zhikang Zhou, Weidong Mi, Li Tong

**Affiliations:** ^1^ Department of Anesthesiology The First Medical Center of Chinese PLA General Hospital Beijing China; ^2^ Department of Anesthesiology The Sixth Medical Center of Chinese PLA General Hospital Beijing China; ^3^ Chinese PLA Medical School Beijing China

**Keywords:** general anesthesia, prelimbic cortex, pyramidal neuron, sevoflurane, ventral tegmental area

## Abstract

**Aims:**

General anesthesia has been used in surgical procedures for approximately 180 years, yet the precise mechanism of anesthetic drugs remains elusive. There is significant anatomical connectivity between the ventral tegmental area (VTA) and the prelimbic cortex (PrL). Projections from VTA dopaminergic neurons (VTA^DA^) to the PrL play a role in the transition from sevoflurane anesthesia to arousal. It is still uncertain whether the prelimbic cortex pyramidal neuron (PrL^Pyr^) and its projections to VTA (PrL^Pyr^‐VTA) are involved in anesthesia‐arousal regulation.

**Methods:**

We employed chemogenetics and optogenetics to selectively manipulate neuronal activity in the PrL^Pyr^‐VTA pathway. Electroencephalography spectra and burst‐suppression ratios (BSR) were used to assess the depth of anesthesia. Furthermore, the loss or recovery of the righting reflex was monitored to indicate the induction or emergence time of general anesthesia. To elucidate the receptor mechanisms in the PrL^Pyr^‐VTA projection's impact on anesthesia and arousal, we microinjected NMDA receptor antagonists (MK‐801) or AMPA receptor antagonists (NBQX) into the VTA.

**Results:**

Our findings show that chemogenetic or optogenetic activation of PrL^Pyr^ neurons prolonged anesthesia induction and promoted emergence. Additionally, chemogenetic activation of the PrL^Pyr^‐VTA neural pathway delayed anesthesia induction and promoted anesthesia emergence. Likewise, optogenetic activation of the PrL^Pyr^‐VTA projections extended the induction time and facilitated emergence from sevoflurane anesthesia. Moreover, antagonizing NMDA receptors in the VTA attenuates the delayed anesthesia induction and promotes emergence caused by activating the PrL^Pyr^‐VTA projections.

**Conclusion:**

This study demonstrates that PrL^Pyr^ neurons and their projections to the VTA are involved in facilitating emergence from sevoflurane anesthesia, with the PrL^Pyr^‐VTA pathway exerting its effects through the activation of NMDA receptors within the VTA.

## Graphical abstract

Our findings revealed that chemogenetic or optogenetic activation of pyramidal neuronal activity in the prelimbic cortex and their projections to the ventral tegmental area prolonged anesthesia induction and promoted emergence. Furthermore, antagonizing NMDA receptors in the ventral tegmental area could reverse the delayed anesthesia induction and promote emergence induced by the activation of pyramidal neurons in the prelimbic cortex projecting to the ventral tegmental area.

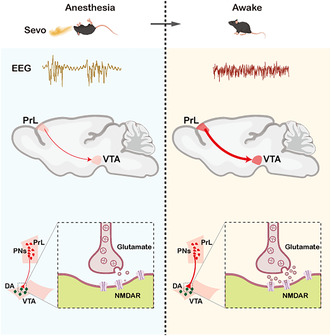

## 1 INTRODUCTION

Nearly 180 years have elapsed since the initial use of general anesthesia in surgical procedures. However, the exact mechanism of general anesthesia remains incompletely understood. Understanding the neurobiological mechanisms of anesthetic induction and recovery of consciousness from anesthesia is critical for precise anesthesia administration, preventing intraoperative awareness, and managing delayed emergence from anesthesia.^1^, ^2^, ^3^ Moreover, investigating the mechanisms of general anesthesia enhances our understanding of consciousness in neuroscience research, with the study of neural circuits playing a pivotal role in decoding these mechanisms.

Research suggests that the central nervous system regulates the transition between anesthesia‐induced unconsciousness and arousal through intricate neural networks.^4^ Nuclei, including the VTA, the lateral hypothalamus, and the locus coeruleus, contribute to anesthesia regulation by projecting to the prefrontal cortex (PFC).^5^, ^6^, ^7^ However, understanding of the anesthetic drugs' mechanisms, based solely on the ascending pathways projecting to the cortex is limited.^8^ Recent advances in cortical research techniques have highlighted the role of intra‐cortical and cortico‐subcortical projections in managing the loss and recovery of consciousness under anesthesia.^9^, ^10^ Nonetheless, these studies primarily focus on disruptions in cortical–cortical and cortical‐thalamic information transmission,^11^, ^12^ as well as alterations in consciousness due to the loss of cortical information integration.^13^, ^14^ The role of descending neural pathways, particularly from the cortex to subcortical nuclei, in the loss and recovery of consciousness remains largely unexplored.

Unlike most cortical regions, the PFC not only receives inputs but also projects them to downstream cell groups, exerting descending control over ascending regulatory systems.^15^ The medial prefrontal cortex (mPFC) is a crucial region for mediating higher cognitive functions and regulating anesthesia‐induced consciousness.^15^ It has extensive neural projections to several arousal‐promoting nuclei in the subcortex.^16^, ^17^ One significant component of the mPFC, the PrL, is closely associated with emotional regulation, decision‐making, and social cognition.^18^, ^19^, ^20^ The VTA maintains extensive functional connections with the PrL, involving reward and motor regulation as well as drug addiction and abuse.^21^, ^22^, ^23^ Previous research has highlighted the significance of the VTA‐PrL neural pathway in the transition from sevoflurane anesthesia to arousal.^24^ Furthermore, studies report that pyramidal neurons in PrL can modulate VTA dopamine neuron activity, and inhibiting PrL^Pyr^ neuron excitatory transmission can reduce morphine‐induced dopamine neuronal activity enhancement in the VTA.^25^ However, the role of PrL^Pyr^ neurons and the PrL^Pyr^‐VTA pathway in regulating anesthesia and arousal remains unclear.

In this study, we selectively activated PrL^Pyr^ neurons and their projections to the VTA by employing transgenic viral transduction, chemogenetics, optogenetics, and pharmacological methods. We investigated the role of PrL^Pyr^ neurons and the PrL^Pyr^‐VTA descending pathway as potential mediators in the transition between anesthesia induction and emergence, utilizing behavioral analysis, electroencephalographic recordings, and spectral analysis. Furthermore, we conducted microinjections of NMDA receptor antagonists (MK‐801) or AMPA receptor antagonists (NBQX) into the VTA, aiming to elucidate the receptor mechanisms by which the PrL^Pyr^‐VTA pathway regulates anesthesia and arousal.

## 2 METHODS

### 2.1 Animals

#### 2.1.1 Experimental animals

Male C57BL/6J mice (8–10 weeks old), weighing 24‐28 g, were obtained from the Experimental Animal Centre of the Chinese People's Liberation Army General Hospital. The mice were housed at the Experimental Animal Facility within the Anaesthesiology Department of the First Medical Centre, Chinese People's Liberation Army General Hospital. The mice were maintained at a room temperature of 22 ± 2°C under a 12 h light/dark cycle (lights on at 7:00 am), with free access to food and water. Behavioral tests were conducted between 09:00 and 18:00 Beijing time. All experimental procedures complied with the Regulations for the Management of Experimental Animals and Animal Experiments. Measurement of loss and recovery of righting reflex and electroencephalogram (EEG) monitoring were conducted 4–6 weeks post‐viral injection. The Animal Ethics Committee approved the experimental protocol (SQ‐2021139), which adhered to our research institution's guidelines for animal experiments.

### 2.2 Stereotaxic surgery

For experimental preparation, the mouse received an intraperitoneal injection of 2% pentobarbital sodium (40–50 mg/kg) for general anesthesia and local anesthesia with 1% lidocaine to the head. Subsequently, the head was immobilized with a stereotaxic apparatus (RWD, Shenzhen, China). Body temperature was maintained at 35–36°C using a heating pad. To validate the function of PrL^Pyr^ neurons, an adeno‐associated viral vector was injected into the PrL at a rate of 30 nL/min using a microinjection pump (Harvard Apparatus, USA). The activation experiment involved unilateral injection, while the inhibition experiment entailed bilateral injection. Stereotaxic reference atlas,^26^ with coordinates referenced to the Bregma. The coordinates were as follows: anterior–posterior (AP) +1.98 mm, medial‐lateral (ML) ±0.25 mm, dorsal‐ventral (DV) −2.1 mm. The total injection volume amounted to 120 nL. Following injection completion, the needle remained in place for 10 min before removal. A 200‐μm diameter ceramic ferrule was positioned 100 μm above the injection site following optogenetic virus injection. For PrL^Pyr^‐VTA neural projection validation, either a microinjection cannula or a ceramic ferrule for optogenetic stimulation was implanted in the VTA. Coordinates were AP −3.4 mm, ML ± 0.3 mm, and DV −4.0 mm. Upon completing the steps, three EEG electrodes were implanted in the skull. The coordinates for the three EEG electrodes were: Electrode 1: AP +1.0 mm, ML −2.0 mm; Electrode 2: AP −4.0 mm, ML −2.5 mm; and Electrode 3: AP −4.0 mm, ML +2.5 mm. Then, the electrodes, optical fibers, or microinjection cannulas were secured to the skull using diluted dental cement.

### 2.3 Anesthesia induction and recovery time calculation

The righting reflex barrel, equipped with an anesthesia gas monitor connected through a circular hole on top (oxygen flow rate: 2 L/min, oxygen concentration: 40%), was used. Initially, mice were allowed to move freely inside the barrel for 15 min. Subsequently, 2.0% sevoflurane inhalation anesthesia was administered. The righting reflex was evaluated by rotating the barrel 180 degrees every 15 s following the induction of anesthesia. If the mouse remained in a dorsally recumbent position (limbs up) and could not spontaneously return to an upright posture, the loss of the righting reflex (LORR) was considered to have occurred, and the time was recorded as the anesthesia induction time. After LORR, sevoflurane anesthesia was maintained by continuous inhalation for 30 min. The vaporizer was then turned off, and the oxygen concentration was maintained while monitoring the righting reflex. The barrel was rotated 180 degrees every 15 s, and if the mouse spontaneously returned to an upright posture (limbs touching the ground), the recovery of the righting reflex (RORR) was considered to have occurred. Anesthesia recovery time was measured from the end of anesthesia to RORR.

### 2.4 EEG recording

Five days after completing the righting reflex experiment, the mice were placed in the barrel and connected to EEG electrodes for monitoring. EEG signals were recorded using a signal amplification system (AD Instruments) and LabChart software (Analog Devices, USA) at a sampling rate of 1000 Hz. For optogenetic EEG activity monitoring, mice were acclimatized in the righting reflex barrel for 15 min. Subsequently, a 5‐min awake baseline was recorded, followed by 30 min of 2.0% sevoflurane inhalation anesthesia, 2 min of photo stimulation occurred during the 25–27 min of the process. Data analysis was conducted using MATLAB (R2020a; MathWorks, USA). Data containing artifacts was excluded, and a filter was employed to eliminate 50 Hz power line interference. Data within the 0.3–50 Hz frequency range were extracted for analysis, focusing specifically on the burst‐suppression ratio before, during, and after the photo stimulation session. For chemogenetic EEG activity monitoring, mice were acclimatized in the righting reflex barrel for 15 min. This was followed by a 5‐min awake baseline recording and 30 min of 2.0% sevoflurane inhalation anesthesia. The burst‐suppression ratio was analyzed for the 5 min before and after the termination of anesthetic delivery. Burst suppression ratio BSR, total power percentage analysis, and spectrum drawing were completed in MATLAB and based on our team's previous studies.^27^, ^28^

### 2.5 Chemogenetic and pharmacology techniques

The chemogenetic virus (rAAV2/9‐CaMKII‐hM3Dq‐mCherry‐WPRE‐pA) or a control virus (rAAV2/9‐CaMKII‐mCherry‐WPRE‐pA) was injected into the PrL region. To validate the PrL^Pyr^‐VTA neural pathway, either the virus (rAAV2/9‐DIO‐hM3Dq‐mCherry‐WPRE‐pA/rAAV2/9‐DIO‐hM4Di‐mCherry‐WPRE‐pA) or the control virus (rAAV2/9‐DIO‐mCherry‐WPRE‐pA) was administered into the PrL region, and another virus (rAAV2/Retro‐CaMKII‐NLS‐Cre) was injected into the VTA region. All viruses were supplied by OBiO Technology Co., Ltd. (Shanghai, China). Following viral injection, microinjection cannulas were implanted in the VTA region to investigate the mechanisms of action of the PrL^Pyr^‐VTA neural pathway receptors. Behavioral tests were conducted 4 weeks after viral transduction and expression. Both the experimental and control groups of mice received an intraperitoneal injection of clozapine N‐oxide (CNO) (MedChemExpress, USA) at a dose of 1 mg/kg. After 45 min, the mice were allowed to acclimatize in the righting reflex barrel for 15 min, followed by 2.0% sevoflurane inhalation anesthesia for 30 min. The durations of loss of righting reflex (LORR) and return of righting reflex (RORR) were documented. For receptor action investigation, DMSO, MK‐801 (an NMDA receptor antagonist, 1.2 nmol/side), and NBQX (an AMPA receptor antagonist, 0.8 nmol/side) (MedChemExpress, USA) were administered via the cannulas. The LORR and RORR durations were recorded after 15 min of drug injection.

### 2.6 Optogenetic technique

For the validation of PrL^Pyr^ or PrL^Pyr^‐VTA projection function, an optogenetic virus (rAAV2/9‐CaMKII‐ChR2‐mCherry‐WPRE‐pA) or a control virus (rAAV2/9‐CaMKII‐mCherry‐WPRE‐pA) was injected into the PrL region. For the validation of PrL^Pyr^ function, a ceramic‐inserted fiber was positioned 100 μm above the virus injection site. For the validation of the PrL^Pyr^‐VTA projections, the virus was injected into the PrL region, and a ceramic‐inserted fiber was inserted into the VTA region. Mice were tested 4 weeks after viral transduction and expression. Before photo stimulation, the mice were allowed to acclimatize in the righting reflex barrel for 15 min, followed by 2.0% sevoflurane inhalation anesthesia for 30 min, and simultaneous blue photo stimulation with a 473‐nm laser (5 mW, 20 Hz, 10 ms duration, 1 s on/1 s off cycle) was applied. Anesthesia induction time was documented. Five days later, the mice were tested for anesthesia recovery time. Mice were anesthetized with 2% sevoflurane for 30 min, after which the vaporizer was turned off and photo stimulation was applied to document the anesthesia recovery time.

### 2.7 Immunofluorescence staining

Following the completion of behavioral experiments, including righting reflex and EEG monitoring, mice were deeply anesthetized using sevoflurane and subsequently perfused with 4% paraformaldehyde (PFA), followed by 0.9% saline. The brain slices were washed three times (5 min each) with 1× PBS to remove OCT. Subsequently, they were incubated with BSA at room temperature for 30 min. After that, the diluted primary antibodies, including glutamate antibody (1:1000, Sigma, G6642, rabbit) and Anti‐c‐fos antibody (1:1000, Abcam, ab208942, mouse), were applied and incubated overnight at 4°C. The slices underwent three 5‐min washes with 1× PBS to remove the primary antibodies. Secondary antibodies (1:500, labeled with Alexa Fluor 488 goat anti‐mouse IgG, Alexa Fluor 488 goat anti‐rabbit IgG, Cy3‐labeled donkey anti‐mouse IgG, Cy3‐labeled donkey anti‐rabbit IgG from Shanghai Beyotime Biotechnology Co., Ltd.) were added and incubated in the dark at room temperature for 2 h. After another wash with 1× PBS, the slices were covered with DAPI and incubated for 15 min. Finally, the images were captured using a fluorescence microscope (Revolution, Echo, America). Experimenters conducting the microscopic analysis were blinded to the group assignments.

### 2.8 Statistical analysis

We used GraphPad Prism 8.0 software (GraphPad, USA) and MATLAB (Mathwork, US) for statistical analysis and graphing. Sample size was referenced from related literature in the field of this research, with 6–8 mice in each group.^28^, ^29^ Data were presented as mean ± standard deviation. The normality of data distributions was tested by the Shapiro‐Wilk test. Two‐tailed Student's *t*‐tests were used for comparing two groups, including righting reflex and immunostaining. Multiple *t*‐tests were used for EEG spectrum analysis. One‐way analysis of variance (ANOVA) followed by Bonferroni's multiple comparison tests was used for pharmacological experiments. A two‐way ANOVA followed by post‐hoc Tukey's multiple comparison tests, was used for measuring BSR per minute during anesthesia. Statistical significance levels are indicated as follows: *p < 0.05, **p < 0.01, and ***p < 0.001.

## 3 RESULTS

### 3.1 Chemogenetic activation of PrL^Pyr^ neurons prolongs anesthesia induction and promotes anesthesia emergence

**FIGURE 1:**
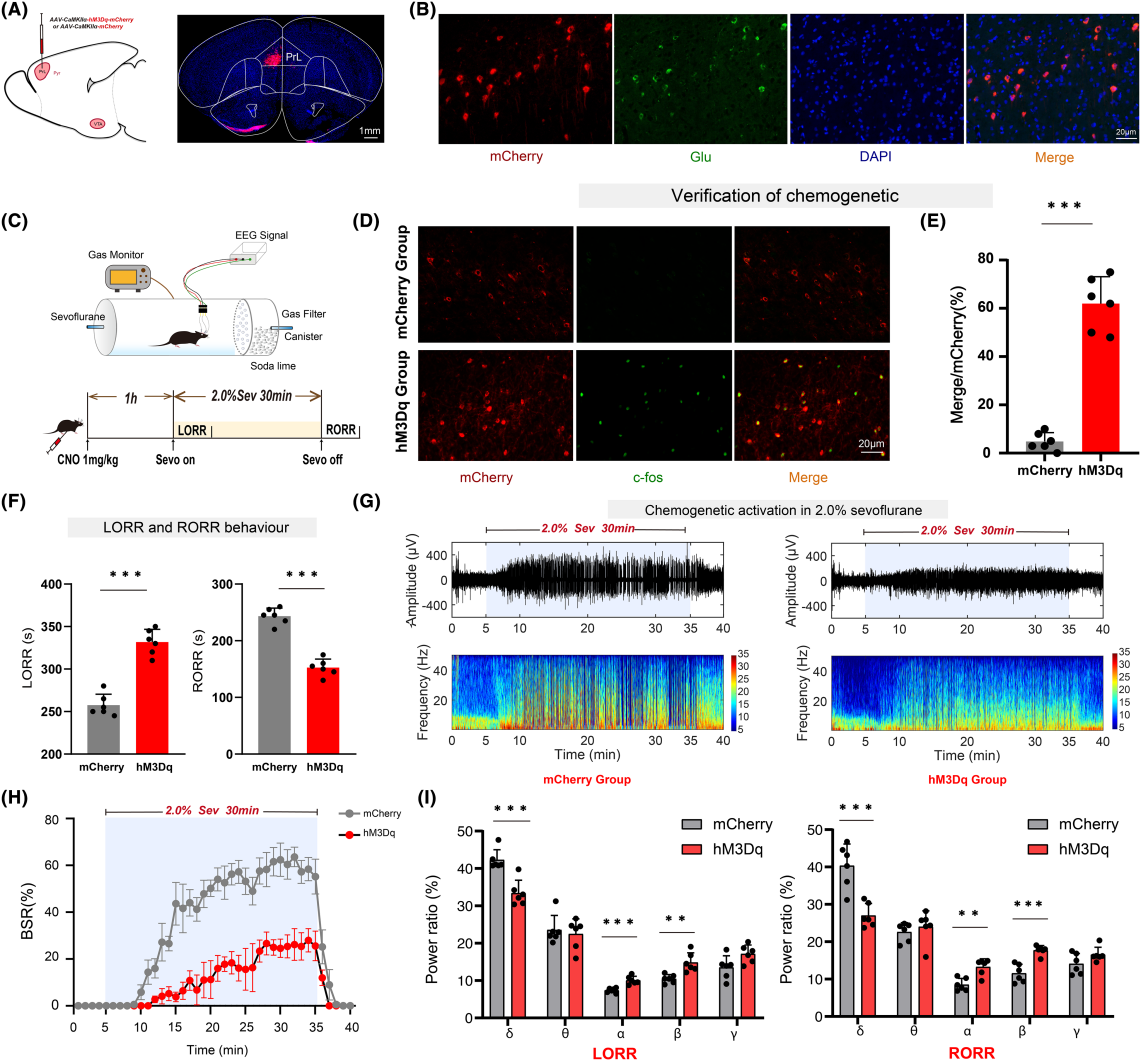
Chemogenetic activation of PrL^Pyr^ neurons prolongs anesthesia induction and promotes anesthesia emergence. (A) An illustration of a virus injection and a coronal brain section demonstrating virus expression in the PrL. (B) The PrL was co‐labeled with mCherry red fluorescence, Glu green immunofluorescence, and DAPI. (C) The process of detecting righting reflexes in anesthesia barrels and recording the EEG signal. (D) Representative immunofluorescent images of double staining of mCherry and c‐fos in the mCherry group and the hM3Dq group. (E) The percentage of c‐fos positive cells was significantly higher in the hM3Dq group (62 ± 11.12%) compared to the mCherry group (4.83 ± 3.656%, *p* < 0.001, *t* = 11.96, *n* = 6) (unpaired *t*‐test). (F) Compared with the mCherry group, the hM3Dq group exhibited a longer mean anesthesia induction time (*p* < 0.001, *t* = 9.15) and a shorter mean anesthesia emergence time (*p* < 0.001, *t* = 10.68, *n* = 6) (unpaired *t*‐test). (G) Two typical EEG spectra for groups of mCherry and hM3Dq. (H) Activation of PrL^Pyr^ neurons significantly decreased the BSR during anesthesia maintenance [*F* (10,390) = 27.47, *p* < 0.001, *n* = 6]. (I) Left, during the process of LORR, the activation of hM3Dq by CNO leads to a reduction in the proportion of power in the δ frequency range (hM3Dq vs. mCherry: 33.39 ± 3.449% vs. 42.31 ± 2.693%, *p* < 0.001, *t* = 4.99, *n* = 6) while increasing it in the α (hM3Dq vs. mCherry: 10.06 ± 1.084% vs. 7.40 ± 0.617%, *p* < 0.001, *t* = 5.24, *n* = 6) and β (hM3Dq vs. mCherry: 14.87 ± 2.575% vs. 10.47 + 1.130%, *p* = 0.0033, *t* ratio = 3.84, *n* = 6) frequency ranges; Right, during the process of RORR, the activation of hM3Dq by CNO leads to a reduction in the proportion of power in the δ frequency range (hM3Dq vs. mCherry: 27.04 ± 3.189% vs. 40.34 ± 5.818%, *p* < 0.001, *t* ratio = 4.91, *n* = 6) while increasing it in the α (hM3Dq vs. mCherry: 13.27 ± 2.149% vs. 8.51 ± 1.691%, *p* = 0.0016, *t* ratio = 4.27, *n* = 6) and β (hM3Dq vs. mCherry: 17.72 ± 1.248% vs. 11.55 ± 2.424% *p* < 0.001, *t* ratio = 5.54, *n* = 6) frequency ranges (Multiple *t*‐test). Data are the mean ± SD (*p < 0.05, **p < 0.01, and ***p < 0.001).

Adeno‐associated viruses were microinjected into the PrL region to selectively manipulate PrL^Pyr^ neurons (Figure 1A, left). Four weeks post‐virus injection, to further investigate the effects of activating PrL^Pyr^ neurons on sevoflurane anesthesia induction and emergence, mice were intraperitoneally injected with CNO. One hour later, the righting reflex test or EEG monitoring was conducted (Figure 1C). Compared to the mCherry group, the hM3Dq group exhibited a significantly prolonged anesthesia induction time and a significantly shortened emergence time (Figure 1F, *p* < 0.001). EEG analysis revealed a significantly reduced burst suppression ratio in the hM3Dq group compared to the mCherry group (Figure 1G,H, *p* < 0.001). During the LORR process, EEG spectral analysis showed that the percentages of power in the δ bands decreased in the hM3Dq group (*p* < 0.001), while those in the α and β bands increased in the hM3Dq group (α, *p* < 0.001; β, *p* = 0.0033, Figure 1I, left). During the RORR process, the percentages of power in the δ bands decreased in the hM3Dq group (*p* < 0.001), while those in the α and β bands increased in the hM3Dq group (α, *p* = 0.0016; β, *p* < 0.001, Figure 1I, right). Following all behavioral experiments, and 1 h after the intraperitoneal administration of CNO, the mice were euthanized, and their brain sections underwent immunostaining; widespread mCherry fluorescence was observed in the PrL region (Figure 1A, right), indicating successful viral expression in pyramidal neurons (Figure 1B). C‐fos immunofluorescence staining was performed on PrL region brain sections (Figure 1D). The results showed a significant increase in c‐fos expression in the PrL region of the hM3Dq group compared to the mCherry group (Figure 1E, *p* < 0.001, Table S1). This indicates that the activity of PrL^Pyr^ neurons was enhanced in the hM3Dq group. These behaviors and EEG findings suggest that the induction and emergence processes of sevoflurane anesthesia are regulated by PrL^Pyr^ neurons.

### 3.2 Optogenetic activation of PrL^Pyr^ neurons delays anesthesia induction and promotes anesthesia emergence

**FIGURE 2:**
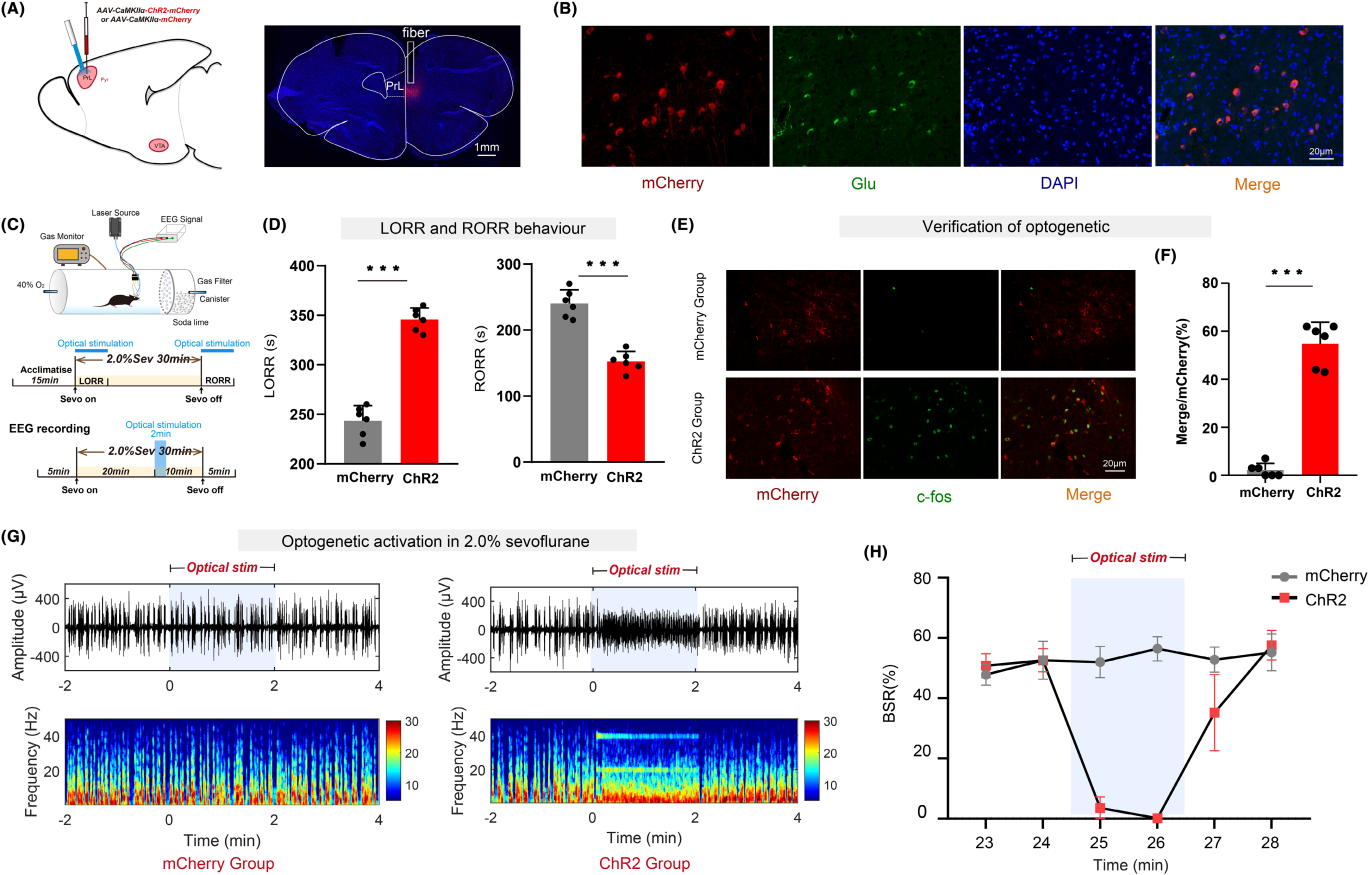
Optogenetic activation of PrL^Pyr^ neurons delays anesthesia induction and promotes anesthesia emergence. (A) Illustration of virus injection and a coronal brain section illustrating virus expression in the PrL. (B) The PrL was co‐labeled with mCherry red fluorescence, Glu green immunofluorescence, and DAPI. (C) The process of detecting righting reflexes in anesthesia barrels and recording the EEG signal. (D) Compared with the mCherry group, the ChR2 group exhibited a longer mean anesthesia induction time (*p* < 0.001, *t* = 13.04) and a shorter mean anesthesia emergence time (*p* < 0.001, *t* = 8.29, *n* = 6) (unpaired *t*‐test). (E) Representative immunofluorescent images of double staining of mCherry and c‐fos in the mCherry group and the ChR2 group. (F) The percentage of c‐fos positive cells was significantly increased in the ChR2 group (54.83 ± 8.909%), compared to the mCherry group (2.17 ± 2.787%, *p* < 0.001, *t* = 13.82, *n* = 6) (unpaired *t*‐test). (G) Two typical EEG spectra for groups of mCherry and ChR2. (H) Activation of PrL^Pyr^ neurons by optogenetic technique (continuous photo stimulation for 2 min) significantly decreased the BSR during anesthesia maintenance in the 25th minute (ChR2 vs. mCherry: 2.62 ± 1.521% vs. 52.01 ± 5.102%) and in the 26th minute (ChR2 vs. mCherry: 0.22 ± 0.415% vs. 56.42 ± 4.106%) ((F5,60) = 101.5, *p* < 0.001, *n* = 6). Data are the mean ± SD; (*p < 0.05, **p < 0.01, and ***p < 0.001).

We performed microinjections of adeno‐associated viruses expressing ChR2 into the PrL region to selectively manipulate the activity of PrL^Pyr^ neurons (Figure 2A, left). Four weeks post‐virus injection, to investigate the effects of optogenetic activation of PrL^Pyr^ neurons on induction and emergence from sevoflurane anesthesia, mice were connected to an optical fiber, placed in a righting reflex barrel, and underwent the righting reflex test or EEG monitoring (Figure 2C). Compared to the mCherry group, the induction time was prolonged, and the emergence time was significantly shortened in the ChR2 group (Figure 2D, *p* < 0.001). EEG monitoring revealed a significant reduction in the burst suppression ratio within 2 min of photo stimulation in the ChR2 group compared to the mCherry group (Figure 2G,H, *p* < 0.001). After EEG monitoring, mice were euthanized and subjected to immunofluorescence staining; widespread mCherry fluorescence was observed in the PrL region (Figure 2A, right), indicating successful viral expression in pyramidal neurons (Figure 2B). C‐fos immunofluorescence staining was performed on PrL region brain sections (Figure 2E). The results showed a significant increase in c‐fos expression in the PrL region of the ChR2 group compared to the mCherry group (Figure 2F, *p* < 0.001, Table S2). This indicates that the activity of PrL^Pyr^ neurons was enhanced in the ChR2 group. These results suggest that the induction and emergence processes of sevoflurane anesthesia are regulated by PrL^Pyr^ neurons.

### 3.3 Chemogenetic activation of the PrL^Pyr^‐VTA neural pathway delays anesthesia induction and promotes anesthesia emergence

**FIGURE 3:**
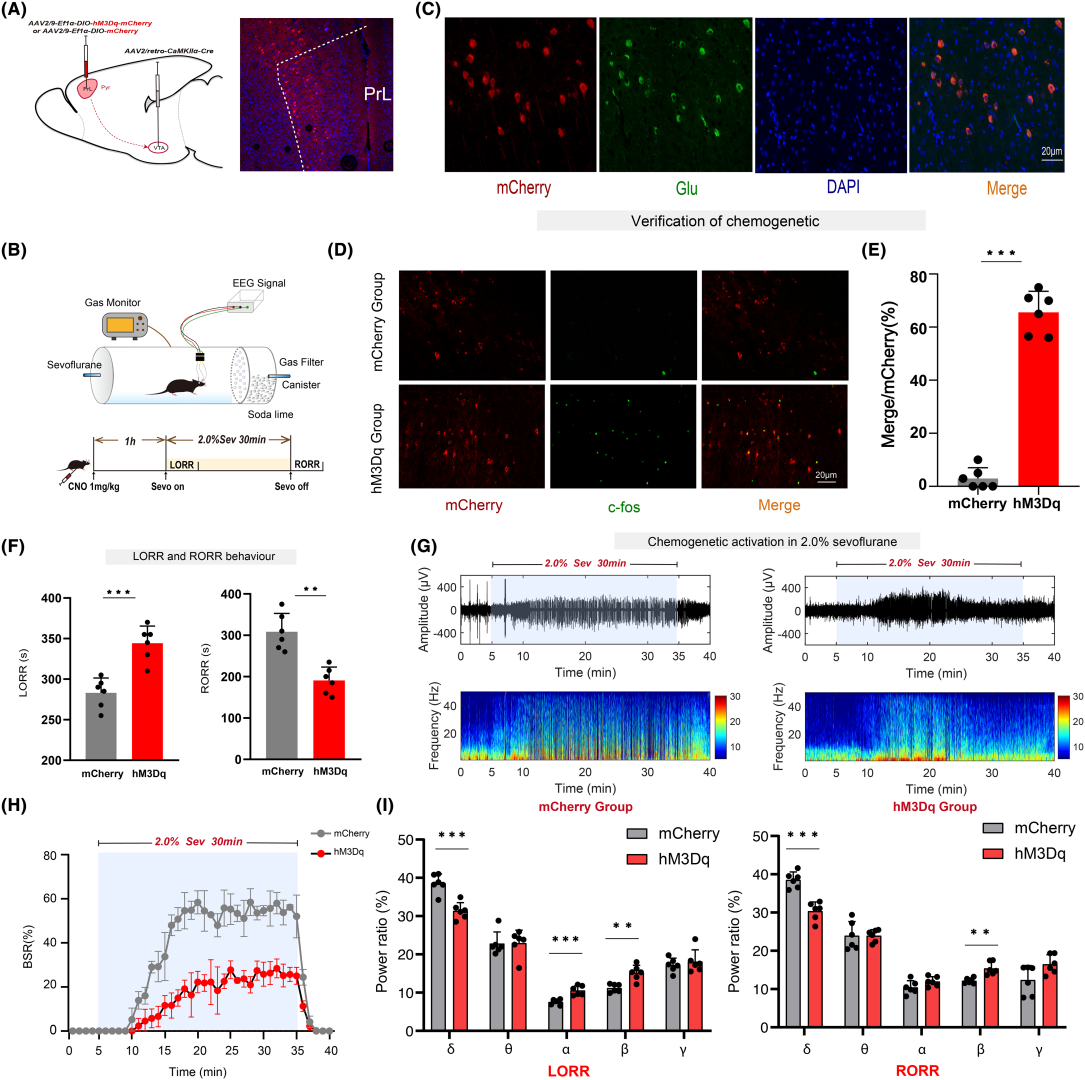
Chemogenetic activation of the PrL^Pyr^‐VTA neural pathway delays anesthesia induction and promotes anesthesia emergence. (A) Illustration of virus injection and a coronal brain section illustrating virus expression in the PrL. (B) The process of detecting righting reflexes in anesthesia barrels and recording the EEG signal. (C) The PrL was co‐labeled with mCherry red fluorescence, Glu green immunofluorescence, and DAPI. (D) Representative immunofluorescent images of double staining of mCherry and c‐fos in the mCherry group and hM3Dq group. (E) The percentage of c‐fos‐positive cells was significantly increased in the hM3Dq group (65.58 ± 7.902%), compared to the mCherry group (3.00 ± 4.00%, *p* < 0.001, *t* = 17.31, *n* = 6) (unpaired *t*‐test). (F) Compared with the mCherry group, the hM3Dq group exhibited a longer mean anesthesia induction time (*p* < 0.001, *t* = 5.32) and a shorter mean anesthesia emergence time (*p* = 0.0012, *t* = 4.48 *n* = 6) (unpaired *t*‐test). (G) Two typical EEG spectra for the mCherry and hM3Dq groups. (H) Activation of PrL^Pyr^‐VTA neurons significantly decreased the BSR during anesthesia maintenance [*F* (10,390) = 35.47, *p* < 0.001, *n* = 6]. (I) Left, during the process of LORR, the activation of hM3Dq by CNO leads to a reduction in the proportion of power in the δ frequency range (hM3Dq vs. mCherry: 31.35 ± 2.185% vs. 38.57 ± 2.455%, *p* < 0.001, *t* ratio = 5.38, *n* = 6) while increasing it in the α (hM3Dq vs. mCherry: 10.55 ± 1.242% vs. 7.57 ± 0.687%, *p* < 0.001, *t* ratio = 5.13, *n* = 6) and β (hM3Dq vs. mCherry: 15.20 ± 1.963 vs. 11.21 ± 1.038%, *p* = 0.0013, *t* ratio = 4.40, *n* = 6) frequency ranges; Right, during the process of RORR, the activation of hM3Dq by CNO leads to a reduction in the proportion of power in the δ frequency range (hM3Dq vs. mCherry: 30.37 ± 2.408% vs. 38.51 ± 2.090%, *p* < 0.001, *t* ratio = 6.25 *n* = 6) while increasing it in the β (hM3Dq vs. mCherry: 15.41 ± 1.693% vs. 12.16 + 0.713%, *p* = 0.0015, *t* ratio = 4.33, *n* = 6) frequency ranges (Multiple *t*‐test). Data are the mean ± SD; (*p < 0.05, **p < 0.01, and ***p < 0.001).

The PrL region was targeted with injections of adeno‐associated viruses expressing either the hM3Dq receptor or control viruses. Additionally, retrograde viruses (rAAV2/Retro‐CaMKII‐NLS‐Cre) were injected into the VTA region. This approach allowed for the selective manipulation of the PrL^Pyr^‐VTA neural pathway (Figure 3A, left). Four weeks after virus injection, to further investigate the effects of activating the PrL^Pyr^‐VTA neural pathway on sevoflurane anesthesia induction and emergence, mice were intraperitoneally injected with CNO. After 1 h, the righting reflex test or EEG monitoring was conducted (Figure 3B). Compared to the mCherry group, the hM3Dq group exhibited a significantly prolonged anesthesia induction time (Figure 3F, left, *p* < 0.001) and a significantly shortened emergence time (Figure 3F, right, *p* = 0.0012). EEG monitoring showed a significantly reduced burst suppression ratio in the hM3Dq group compared to the mCherry group (Figure 3G,H, *p* < 0.001). During the LORR process, EEG spectral analysis showed that the percentages of power in the δ bands, decreased in the hM3Dq group (*p* < 0.001), while those in the α and β bands increased in the hM3Dq group (α, *p* < 0.001; β, *p* = 0.0013, Figure 3I, left), compared to the mCherry group. During the RORR process, the percentages of power in the δ bands decreased in the hM3Dq group (*p* < 0.001), while those in the β bands increased in the hM3Dq group (*p* = 0.0015, Figure 3I, right), compared to the mCherry group. EEG spectral analysis showed that after PrL^Pyr^‐VTA was activated, the power ratio of the low‐frequency band associated with anesthesia decreased, while the power ratio of the high‐frequency band associated with wakefulness increased. After completion of all behavioral experiments, mice were euthanized, and subjected to immunofluorescence staining, widespread mCherry fluorescence was observed in the PrL region (Figure 3A, right), indicating successful viral expression in pyramidal neurons (Figure 3C). C‐fos immunofluorescence staining was performed on brain sections in the PrL region (Figure 3D). The results showed a significant increase in c‐fos expression in the PrL region of the hM3Dq group compared to the mCherry group (Figure 3E, *p* < 0.001, Table S3). Concurrently, we inhibited this pathway via chemogenetics, which can promote anesthesia induction and delay anesthesia emergence (see Figure S1). These behaviors and EEG findings suggest that the induction and emergence processes of sevoflurane anesthesia are regulated by the PrL^Pyr^‐VTA neural pathway.

### 3.4 Optogenetic activation of the PrL^Pyr^‐VTA neural pathway delays anesthesia induction and promotes anesthesia emergence

**FIGURE 4:**
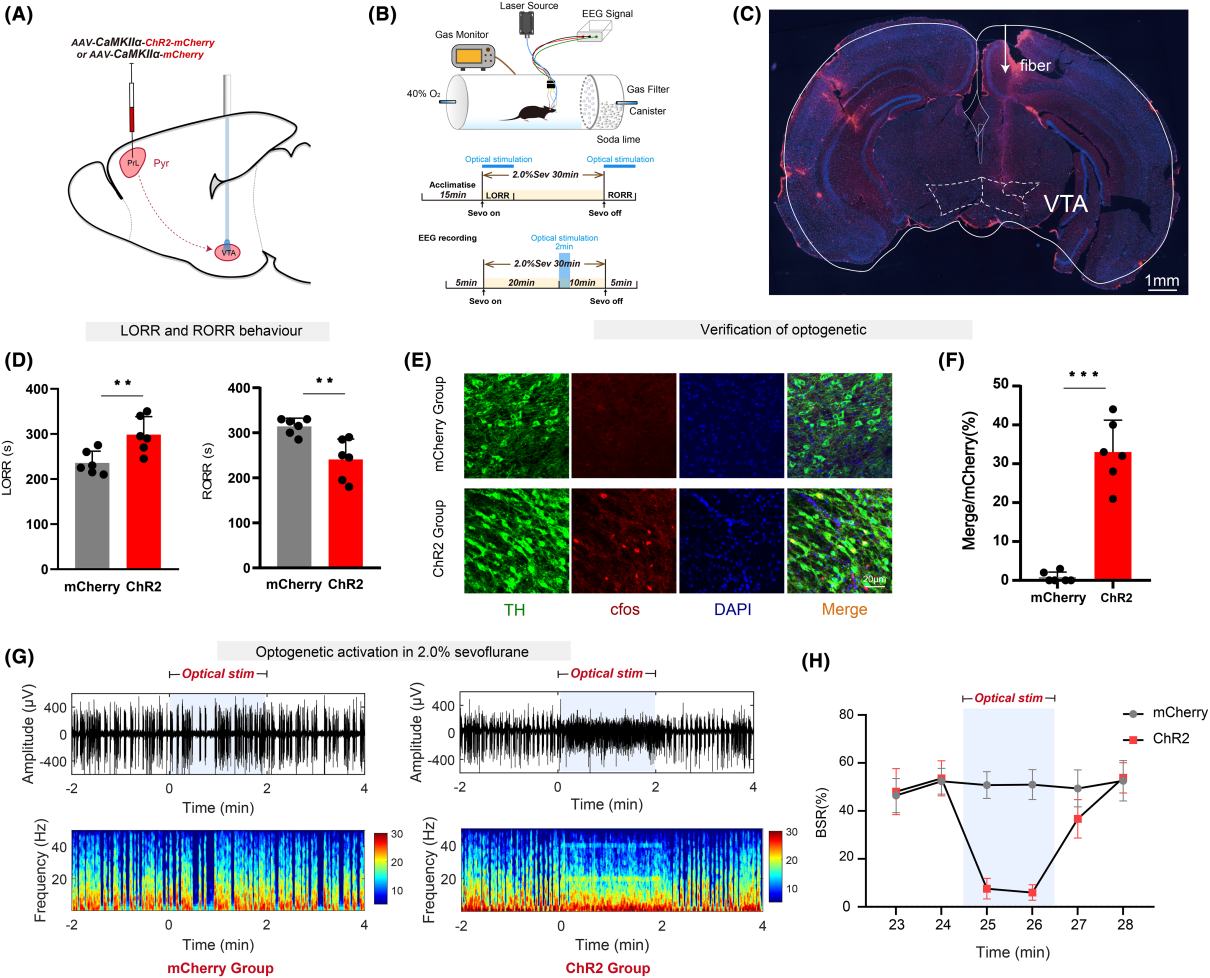
Optogenetic activation of the PrL^Pyr^‐VTA neural pathway delays anesthesia induction and promotes anesthesia emergence. (A) The schematic depicts the injection of a virus into the PrL region while an optical fiber is implanted in the VTA area. (B) The process of detecting righting reflexes in anesthesia barrels and recording the EEG signal. (C) Representative brain slice with VTA optical fiber implantation. (D) Compared with the mCherry group, the ChR2 group exhibited a longer mean anesthesia induction time (*p* = 0.0096, *t* = 3.19) and a shorter mean anesthesia emergence time (*p* = 0.0043, *t* = 3.68, *n* = 6) (unpaired *t*‐test). (E) Representative immunofluorescent images of double staining of TH and c‐fos in the mCherry and ChR2 groups. (F) The percentage of c‐fos‐positive cells was significantly increased in the ChR2 group (33.0 ± 3.367%), compared to the mCherry group (0.83 ± 1.329%, *p* < 0.001, *t* = 9.43, *n* = 6) (unpaired *t*‐test). (G) Two typical EEG spectra for the mCherry and ChR2 groups. (H) Activation of PrL^Pyr^‐VTA neuronal pathway by optogenetic technique (continuous photo stimulation for 2 min) significantly decreased the BSR during anesthesia maintenance in the 25th minute (ChR2 vs. mCherry:7.61 ± 4.313% vs. 50.80 ± 5.513%) and in the 26th minute (ChR2 vs. mCherry: 6.02 ± 3.214% vs. 51.0 ± 6.243%) ((F5, 60) = 30.22, *p* < 0.001, *n* = 6). Data are the mean ± SD (*p < 0.05, **p < 0.01, and ***p < 0.001).

The PrL region received injections of adeno‐associated viruses expressing ChR2 or control viruses, while a ceramic implant was surgically placed in the VTA region. This combination allowed for the selective manipulation of the PrL^Pyr^‐VTA neural pathway (Figure 4A). Optogenetic methods were employed to activate the PrL^Pyr^‐VTA neural pathway and observe its effects on sevoflurane anesthesia induction and emergence. Mice were connected to an optical fiber and placed in a righting reflex barrel (Figure 4B). Compared to the mCherry group, the ChR2 group exhibited a significantly prolonged anesthesia induction time (Figure 4D, left, *p* = 0.0096) and a significantly shortened emergence time (Figure 4D, right, *p* = 0.0043). EEG monitoring showed a significantly reduced burst suppression ratio in the ChR2 group during photo stimulation compared to the mCherry group (Figure 4G,H, *p* < 0.001). After completion of all behavioral experiments, mice were euthanized, and the ceramic implant was found to be in the proper position (Figure 4C). Immunofluorescence staining was performed on brain sections in the VTA region (Figure 4E), and the results showed a significant increase in c‐fos expression in the VTA region of the ChR2 group compared to the mCherry group (*p* < 0.001; Figure 4F, Table S4). These behaviors and immunofluorescence staining suggest that the PrL^Pyr^‐VTA neural pathway can regulate the induction and emergence processes of sevoflurane anesthesia by exciting dopaminergic neurons in the VTA region.

### 3.5 Chemogenetic inhibition of the PrL^Pyr^‐VTA neural pathway promotes anesthesia induction and delays anesthesia emergence

**FIGURE 5:**
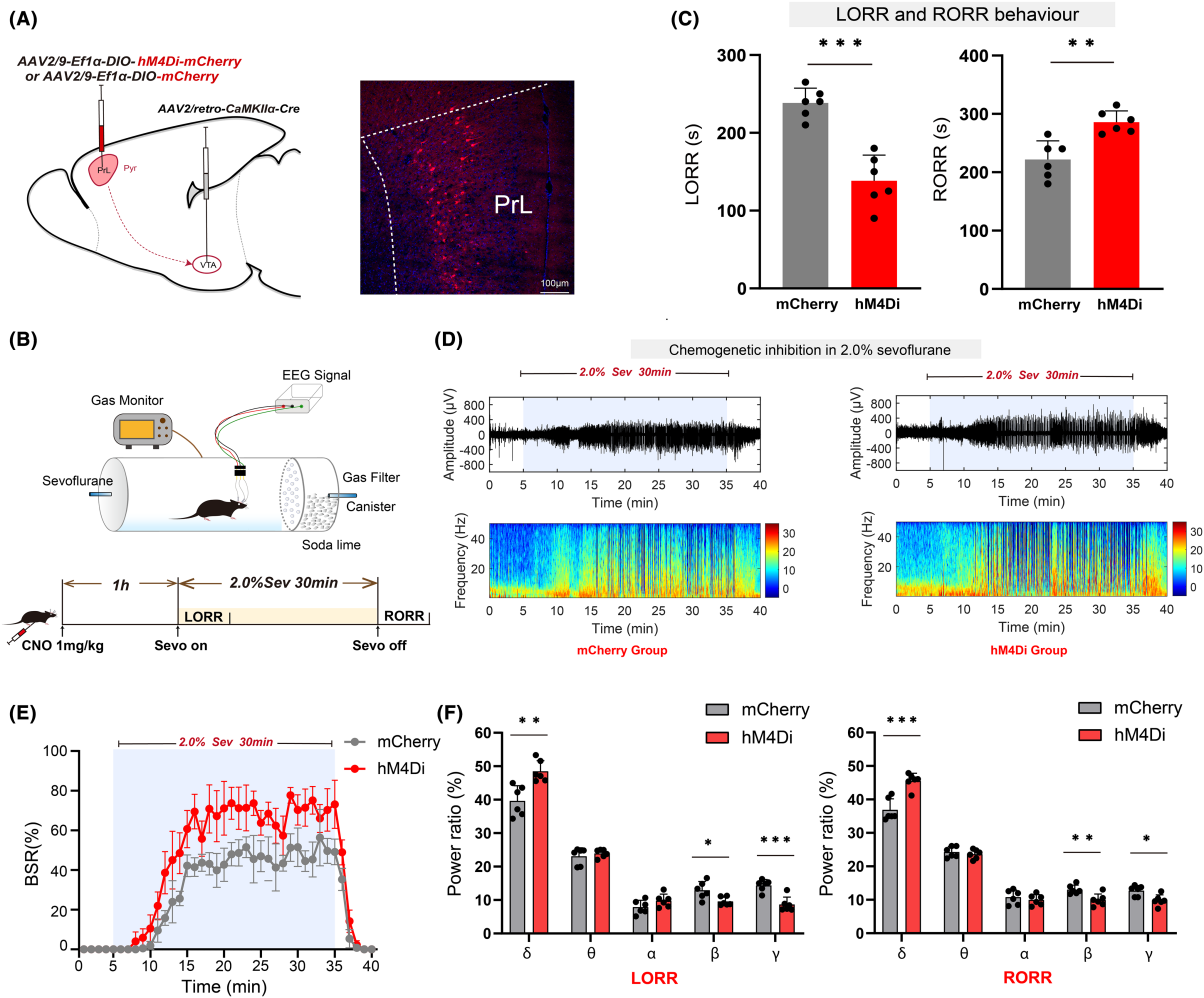
Chemogenetic inhibition of the PrLPyr‐VTA neural pathway promotes anesthesia induction and delays anesthesia emergence. (A) Illustration of virus injection and a coronal brain section illustrating virus expression in the PrL. (B) The process of detecting righting reflexes in anesthesia barrels and recording the EEG signal. (C) Compared to the mCherry group, the hM4Di group exhibited a shorter mean anesthesia induction time (*p* < 0.001, *t* = 6.43) and a longer mean anesthesia emergence time (*p* = 0.0018, *t* = 4.199, *n* = 6) (unpaired *t*‐test). (D) Two typical EEG spectra for the mCherry and hM4Di groups. (E) Inhibition of PrL^Pyr^‐VTA neurons significantly increased the BSR during anesthesia maintenance [*F* (10,390) = 2.372, *p* < 0.001, *n* = 6]. (F) Left, During the process of LORR, inhibition of hM4Di by CNO led to an increase in the proportion of power in the δ frequency range (hM4Di vs. mCherry: 48.47 ± 3.217% vs. 39.65 ± 4.502%, *p* = 0.0029, *t* ratio = 3.90, *n* = 6) while decreasing it in the β (hM4Di vs. mCherry: 9.57 ± 1.265% vs. 12.91 ± 2.611%, *p* = 0.0182, *t* ratio = 2.818, *n* = 6) and γ (hM4Di vs. mCherry: 8.66 ± 2.229 vs. 14.39 ± 1.720%, *p* < 0.001, *t* ratio = 4.99, *n* = 6) frequency ranges; Right, During the process of RORR, inhibition of hM4Di by CNO led to an increase in the proportion of power in the δ frequency range (hM4Di vs. mCherry: 45.54 ± 2.304% vs. 36.88 ± 3.298%, *p* < 0.001, *t* ratio = 5.27 *n* = 6) while decreasing it in the β (hM4Di vs. mCherry: 9.91 ± 1.834% vs. 13.03 ± 1.337%, *p* = 0.0072, *t* ratio = 3.37, *n* = 6) and γ frequency ranges (hM4Di vs. mCherry: 9.82 ± 1.676% vs. 12.72 ± 1.554%, *p* = 0.0111, *t* ratio = 3.11, *n* = 6) (Multiple *t*‐test). Data are the mean ± SD (*p < 0.05, **p < 0.01, and ***p < 0.001).

The PrL region was targeted with injections of either the hM4Di receptor or control adeno‐associated viruses. Additionally, retrograde viruses (rAAV2/Retro‐CaMKII‐NLS‐Cre) were injected into the VTA region, enabling selective manipulation of the PrL^Pyr^‐VTA neural pathway (Figure 5A, left). Four weeks post‐virus injection, to further explore the effects of inhibiting the PrL^Pyr^‐VTA neural pathway on sevoflurane anesthesia induction and emergence, mice received intraperitoneal injections of CNO. One hour later, either the righting reflex test or EEG monitoring was conducted (Figure 5B). Compared to the mCherry group, the hM4Di group exhibited a significantly shortened anesthesia induction time (Figure 5C, left, *p* < 0.001) and a significantly prolonged emergence time (Figure 5C, right, *p* = 0.0018). EEG monitoring revealed a significantly increased burst suppression ratio in the hM4Di group compared to the mCherry group (Figure 5D,E; *p* < 0.001). During the LORR process, EEG spectral analysis indicated an increase in power percentages in the δ bands in the hM4Di group (*p* = 0.0029), while power in the β and γ bands decreased (β, *p* = 0.0182; γ, *p* < 0.001, Figure 5F, left), compared to the mCherry group. During the RORR process, there was an increase in the percentages of power in the δ bands in the hM4Di group (*p* < 0.001), while the percentages in the β and γ bands decreased (β, *p* = 0.0071; γ, *p* = 0.0111; Figure 5F, right) compared to the mCherry group. EEG spectral analysis revealed that inhibition of the PrL^Pyr^‐VTA pathway led to an increase in activity in the low‐frequency band associated with sleep and a decrease in activity in the high‐frequency band associated with wakefulness. Following the completion of all behavioral experiments, the mice were euthanized and subjected to validation of virus expression. Widespread mCherry fluorescence was observed in the PrL region (Figure 5A, right), indicating successful viral expression in PrL. These behaviors and EEG findings suggest that the PrL^Pyr^‐VTA neural pathway regulates the induction and emergence processes of sevoflurane anesthesia.

### 3.6 PrL^Pyr^‐VTA neural pathway exerted its effect mainly through NMDA receptors in VTA

**FIGURE 6:**
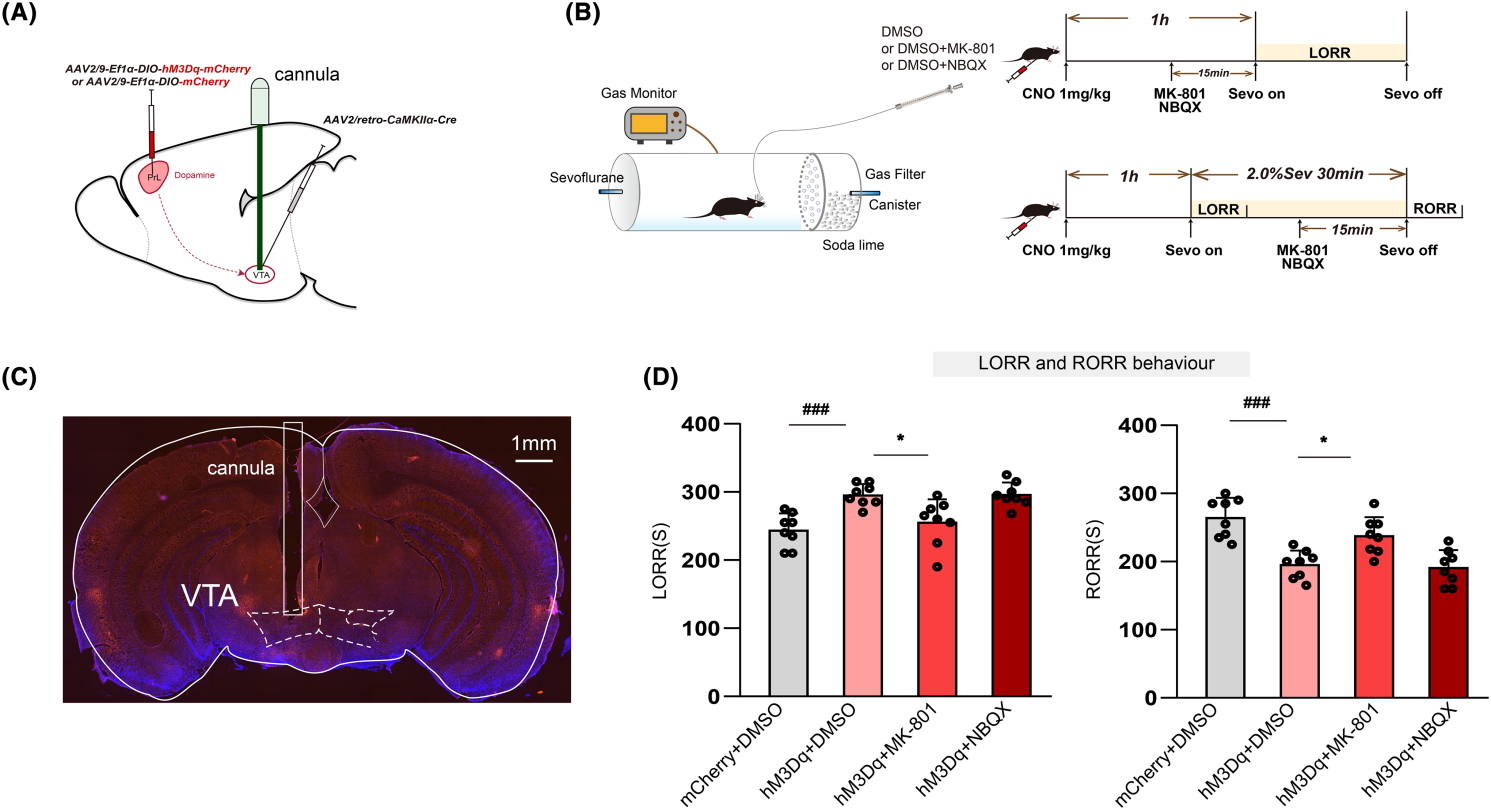
The PrL^Pyr^‐VTA neural pathway exerts its effect mainly through NMDA receptors in VTA. (A) Schematic illustration of virus injection in the PrL and VTA regions, where the microinjection cannulas were implanted in the VTA region. (B) Left, righting reflex detection; right, the process of detecting anesthesia induction and emergence times through chemogenomic activation of the PrL^Pyr^‐VTA neurons and blocking of NMDA/AMPA receptors in 2.0% sevoflurane anesthesia. (C) Representative brain slice with VTA microinjection cannula implantation. (D) Left, the mean anesthesia induction time is longer in the hM3Dq + DMSO group compared to mCherry+DMSO group (*p <* 0.001, *n* = 8), and the induction time is shorter in the hM3Dq + MK‐801 group compared to the hM3Dq + DMSO group (*p* = 0.012, *n* = 8) (*F* (3, 28) = 14.66); Right, the emergence time is shorter in the hM3Dq + DMSO group compared to the mCherry + DMSO group (*p* < 0.001, *n* = 8), and the emergence time is longer in the hM3Dq + MK‐801 group compared to the hM3Dq + DMSO group (*p* = 0.014, *n* = 8) (*F* (3, 20) = 8.373). Data are the mean ± SD; (*p < 0.05, **p < 0.01, and ^###^ *p* < 0.001).

Adeno‐associated viruses carrying the hM3Dq receptor, or control viruses, were injected into the PrL region using a microinjection needle. Additionally, a retrograde adeno‐associated virus (rAAV2/Retro‐CaMKII‐NLS‐Cre) was injected into the VTA region to selectively manipulate the PrL^Pyr^‐VTA neural pathway (Figure 6A). Following 45 min of intraperitoneal administration of CNO to mice, they were placed in a righting reflex barrel. Microinjection cannulas were connected, and either DMSO, MK‐801, or NBQX were injected into the VTA. After a 15‐min acclimation period, sevoflurane anesthesia was initiated at a 2% concentration, and the time to LORR was recorded. The time to emergence from anesthesia was measured, and DMSO, MK‐801, or NBQX were injected using microinjection cannulas after 15 min of anesthesia. The vaporizer was turned off after 30 min of anesthesia, and the time to RORR was recorded (Figure 6B).The results demonstrated that, compared to the mCherry+DMSO group, activation of the PrL^Pyr^‐VTA neural pathway in the hM3Dq + DMSO group led to a delay in anesthesia induction and facilitated anesthesia emergence (Figure 6D, *p* < 0.001). In the hM3Dq + MK‐801 group, administration of MK‐801 in the VTA region significantly attenuated the prolonged anesthesia induction time (Figure 6D, left, *p* = 0.012) and the shortened emergence time compared to the hM3Dq + DMSO group (Figure 6D, right, *p* = 0.014) induced by PrL stimulation. However, in the hM3Dq + NBQX group, administration of NBQX in the VTA region did not significantly impact either anesthesia induction time or emergence time compared to the hM3Dq + DMSO group. After completion of all behavioral experiments, mice were euthanized, and it was confirmed that the microinjection cannula had been accurately implanted into the VTA (Figure 6C). These findings suggest that blocking NMDA receptors in the VTA region can attenuate the delayed anesthesia induction and promote emergence, which is induced by activation of the PrL^Pyr^‐VTA neural pathway.

## 4 DISCUSSION

In this study, we combined chemogenetics and optogenetics with behavioral and EEG monitoring; this approach validated that activating PrL^Pyr^ not only delays the induction of sevoflurane anesthesia but also promotes anesthesia arousal. Additionally, we applied the same methods to confirm that activating the PrL^Pyr^‐VTA neural pathway can slow down the induction of sevoflurane anesthesia and accelerate anesthesia emergence. By employing chemical genetics combined with pharmacology, we demonstrated that the PrL^Pyr^‐VTA neural pathway exerts its effects on promoting anesthesia emergence by acting on NMDA receptors in the VTA region.

Advancements in cortical observation techniques and intracortical electrode recordings have accumulated substantial knowledge about the effects of general anesthesia on the cortex. Another study has highlighted the “fragmentation” of information transmission between cortical regions as a significant factor leading to the loss of consciousness during general anesthesia.^11^ Anesthetics disrupt functional connectivity between cortical regions, impairing the cortex's ability to transmit and integrate information, resulting in the loss of consciousness.^14^, ^30^ Researchers increasingly recognize the importance of the “Top‐Down” mechanism in regulating the loss and recovery of consciousness during general anesthesia, but the cellular and circuit‐level mechanisms underlying this remain unclear.

General anesthetics exhibit varying degrees of inhibition in different regions of the cerebral cortex, with a preference for inhibiting neurons in higher‐order cortical regions while relatively preserving neurons in lower‐level sensory areas.^31^ The mPFC is a crucial brain region involved in regulating higher‐order functions like emotion, cognition, decision‐making, and consciousness.^32^ A study found that delivery of cholinergic agonists into the mPFC during sevoflurane anesthesia can induce wakefulness, highlighting the mPFC's role in anesthesia regulation.^3^ Deactivating the mPFC delays the recovery of consciousness under general anesthesia.^33^ Specific excitation of the mPFC‐dorsal medial thalamus circuit can reduce anesthesia depth and promote awakening, suggesting the mPFC's importance as a key downstream nucleus in the “Top‐Down” regulation of general anesthesia.^34^ The PrL, a subregion of the mPFC, is closely connected to subcortical regions and plays a crucial role in cognitive functions and emotional regulation.^35^, ^36^ However, its involvement in anesthesia‐emergence processes has been understudied. In our previous study, we found that dopamine neurons in the VTA‐PrL pathway exert a wake‐promoting effect during sevoflurane anesthesia via the D1 receptor.^24^ One study reported that inactivation of PrL attenuates behavioral arousal induced by stimulation of the basal forebrain during sevoflurane anesthesia.^37^ These findings highlight the importance of the PrL in the anesthesia‐emergence process. However, the downstream regulatory mechanisms of the PrL and the mechanisms by which its projections mediate the anesthesia‐emergence transition remain unclear.

The VTA, a target nucleus for cortical downstream regulation, has wake‐promoting effects and contains DA and GABAergic neurons; it receives inputs from pyramidal neurons in the PFC^40^. It plays a crucial role in processes such as reward, learning, memory, motivation, and anesthesia.^38^, ^39^, ^40^, ^41^ By projecting to the VTA, the PFC can modulate VTA neuron activity, influencing behavioral choices and reward learning processes. NMDA receptors, the major excitatory glutamate receptors in the central nervous system, have been linked to the neuroprotective effects of inhaled anesthetics. The application of NMDA antagonists in the central nervous system can antagonize thiopental sodium‐induced anesthesia,^42^ suggesting their involvement in the actions of anesthetics and as important targets for general anesthesia. In our study, blocking NMDA receptors in the VTA region counteracted the wake‐promoting effects of upstream PrL^Pyr^ neurons, while blocking AMPA receptors had no such effect. This indicates that the PrL^Pyr^‐VTA pathway regulates the anesthetic effects of sevoflurane through NMDA receptors. Our previous study found that activating VTA^DA^‐PrL facilitates the emergence of sevoflurane anesthesia,^24^ and the present study found that the PrL^Pyr^‐VTA pathway also plays a role in promoting anesthesia arousal. The involvement of bidirectional projection implies that the feedforward‐feedback loop may have a crucial impact on anesthesia‐awakening.^9^ Further investigations into the role of the forward‐feedback loop in the mechanism of general anesthesia can be conducted in future studies. which may reveal how conscious cognitive information is processed and suggest that mechanisms for maintaining arousal should be further explored. The PrL also has direct reciprocal connections with other arousal‐promoting subcortical nuclei, such as the basal forebrain and locus coeruleus.^37^, ^43^ The arousal‐promoting effect of the PrL could also be mediated through these nuclei, necessitating further exploration of these interactions. The limitation of the present study is that neurons transfected with the CaMKII promoter contain a wide range of pyramidal neuron subtypes. Diverse subtypes of pyramidal neurons in the cortex have been identified.^44^ The specific subtype of PrL^Pyr^ neurons involved in regulating anesthesia arousal remains unclear in our study. Another limitation of our study is the exclusive use of male mice, which may not entirely represent the responses that could be observed in female mice. Including both sexes in future research would afford a more comprehensive understanding of the findings.

## 5 CONCLUSION

In conclusion, this study demonstrated that the PrL^Pyr^‐VTA circuit is a central element in regulating the mechanism of general anesthesia. Furthermore, inhibition of NMDA receptors could attenuate the delayed anesthesia induction and wakefulness‐promoting effect elicited by activation of the PrL^Pyr^‐VTA circuit. These findings enhance our understanding of descending neural circuits in the regulation of consciousness during general anesthesia.

## AUTHOR CONTRIBUTIONS

Fuyang Cao, Yongxin Guo, and Shuting Guo contribute equally to the manuscript. Fuyang Cao, Yongxin Guo, and Shuting Guo interpreted the data, completed the virus injection, immunofluorescence staining, and drafted the manuscript. Weidong Mi and Li Tong contributed to the study's conception and design, provided technical or material support, and made critical revisions of the manuscript. Fuyang Cao, Xinyu Hao, Lujia Yang, and Jiangbei Cao did the statistical analysis and creation of figures. Zhikang Zhou and Xinyu Hao completed the righting reflex test and EEG recording.

## FUNDING INFORMATION

This work was supported by the National Natural Science Foundation of China (Grant No. 81801366 to Li Tong, Grant No. 82001453 to Yongxin Guo, and Grant No. 82171180 to Weidong Mi).

## CONFLICT OF INTEREST STATEMENT

The authors state that there are no conflicts of interest to disclose.

## Supporting information


Figure S1.

## Data Availability

The datasets analyzed during the study are available from the corresponding author on reasonable request.
