# Supplementary material for: Prelimbic cortical pyramidal neurons to ventral tegmental area projections promotes arousal from sevoflurane anesthesia
Source: CNS Neurosci Ther. 2024 Mar 15;30(3):e14675. doi: 10.1111/cns.14675 (PMC10941502; doi:10.1111/cns.14675)
Supplement: Supplementary file 1 — Figure S1. [file CNS-30-e14675-s001.docx]

**Supplementary materials**

**RESULTS**

**Chemogenetic inhibition of the PrL^Pyr^-VTA neural pathway promotes anaesthesia induction and delays anaesthesia emergence.**

The PrL region was targeted with injections of expressing either the hM4Di receptor or control adeno-associated viruses. Additionally, retrograde viruses (rAAV2/Retro-CaMKII-NLS-Cre) were injected into the VTA region, enabling selective manipulation of the PrL^Pyr^-VTA neural pathway (Fig. S1, A, left). Four weeks post-virus injection, to further explore the effects of inhibiting the PrL^Pyr-^VTA neural pathway on sevoflurane anaesthesia induction and emergence, mice received intraperitoneal injections of CNO. One hour later, either the righting reflex test or EEG monitoring was conducted (Fig. S1.B). Compared to the mCherry group, the hM4Di group exhibited a significantly shortened anaesthesia induction time (Fig. S1, left, P<0.001) and a significantly prolonged emergence time (Fig. S1, right, P=0.0018). EEG monitoring revealed a significantly increased burst suppression ratio in the hM4Di group compared to the mCherry group (Fig. S1, D, E; P<0.001). During the LORR process, EEG spectral analysis indicated an increase in power percentages in the δ bands in the hM4Di group (P=0.0029), while power in the β and γ bands decreased (β, P=0.0182; γ, P<0.001, Fig. S1, F, left), compared to the mCherry group. During the RORR process, there was an increase in the percentages of power in the δ bands in the hM4Di group (P<0.001), while the percentages in the β and γ bands decreased (β, P=0.0071; γ, P=0.0111; Fig. S1, F, right) compared to the mCherry group. EEG spectral analysis revealed that inhibition of the PrL^Pyr^-VTA pathway led to an increase in activity in the low-frequency band associated with sleep and a decrease in activity in the high-frequency band associated with wakefulness. Following the completion of all behavioural experiments, the mice were euthanised and subjected to validation of virus expression. Widespread mCherry fluorescence was observed in the PrL region (Fig. S1, A, right), indicating successful viral expression in PrL. These behaviours and EEG findings suggest that the PrL^Pyr^-VTA neural pathway regulates the induction and emergence processes of sevoflurane anaesthesia.


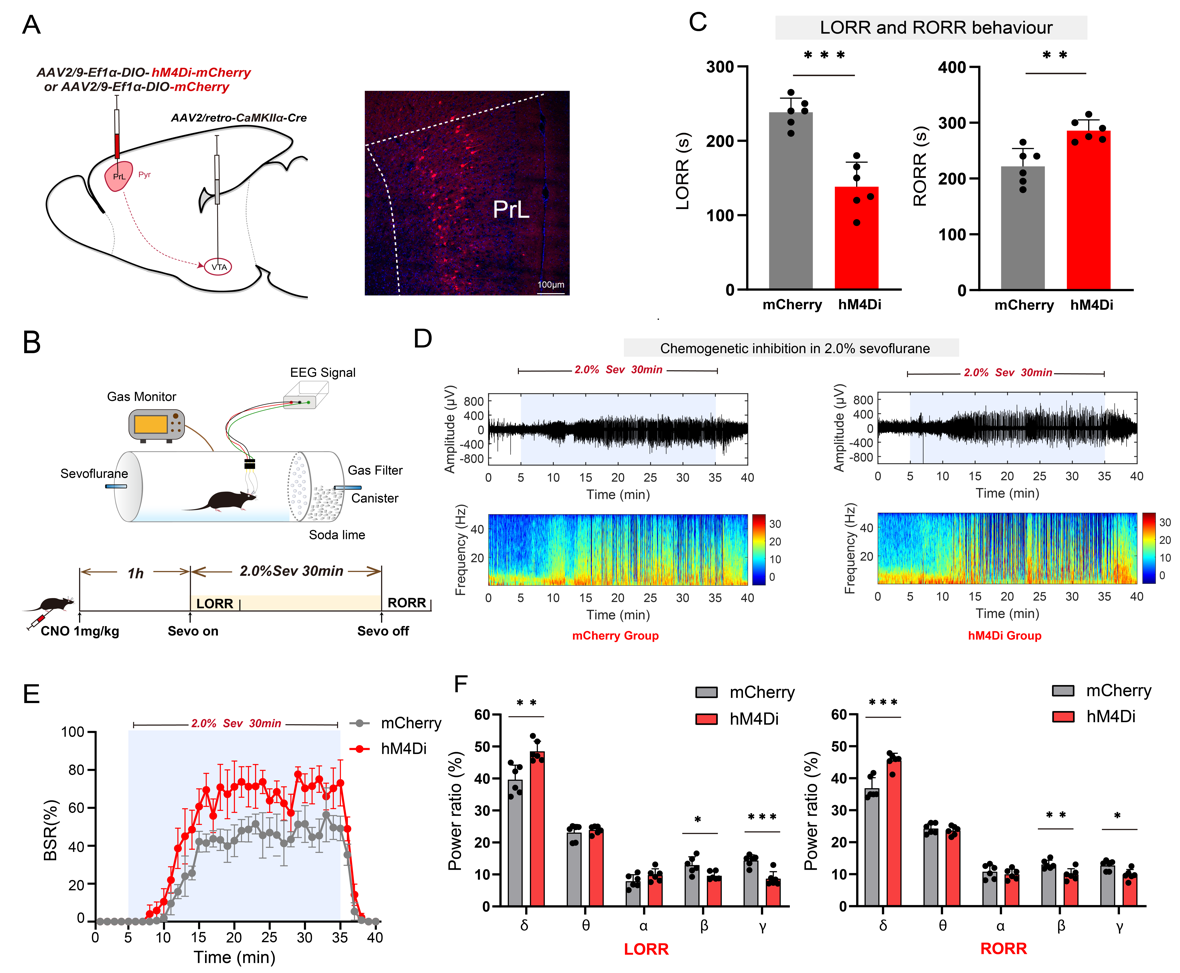


**FIGURE S1** Chemogenetic inhibition of the PrL^Pyr^-VTA neural pathway promotes anaesthesia induction and delays anaesthesia emergence. A, Illustration of virus injection and a coronal brain section illustrating virus expression in the PrL. B, The process of detecting righting reflexes in anaesthesia barrels and recording the EEG signal. C, Compared to the mCherry group, the hM4Di group exhibited a shorter mean anaesthesia induction time (P<0.001, t=6.43) and a longer mean anaesthesia emergence time (P=0.0018, t=4.199 n = 6) (unpaired t-test) D, Two typical EEG spectra for the mCherry and hM4Di groups. E, Inhibition of PrL^Pyr^-VTA neurons significantly increased the BSR during anaesthesia maintenance [F (10,390) =2.372, P<0.001, n=6]. F, Left, During the process of LORR, inhibition of hM4Di by CNO led to an increase in the proportion of power in the δ frequency range (hM4Di vs mCherry: 48.47± 3.217% vs 39.65 ± 4.502%, P=0.0029, t ratio=3.90, n = 6) while decreasing it in the β (hM4Di vs mCherry : 9.57 ± 1.265% vs 12.91 ± 2.611%, P=0.0182, t ratio=2.818, n = 6) and γ (hM4Di vs mCherry: 8.66±2.229 vs 14.39±1.720%, P<0.001, t ratio =4.99, n = 6) frequency ranges; Right, During the process of RORR, inhibition of hM4Di by CNO led to an increase in the proportion of power in the δ frequency range (hM4Di vs mCherry: 45.54 ± 2.304% vs 36.88 ± 3.298%, P<0.001, t ratio=5.27 n = 6) while decreasing it in the β (hM4Di vs mCherry: 9.91 ± 1.834% vs 13.03± 1.337%, P=0.0072, t ratio=3.37, n = 6) and γ frequency ranges (hM4Di vs mCherry: 9.82±1.676% vs 12.72±1.554%, P=0.0111, t ratio =3.11, n = 6) (Multiple t-test). Data are the mean ± SD; (*P < 0.05, **P < 0.01, and ***P < 0.001).

**Supplementary tables to show how many neurons were counted in each group.**

**Table S1 Chemogenetic activation of PrL^Pyr^ neurons prolongs anaesthesia induction and promotes anaesthesia emergence.**

| **Group**  **Type**  **Number** | **mCherry** | | **hM3Dq** | |
| --- | --- | --- | --- | --- |
|  | **c-fos** | **neurones** | **c-fos** | **neurones** |
| 1 | 2 | 26 | 18 | 29 |
| 2 | 1 | 30 | 17 | 26 |
| 3 | 1 | 22 | 18 | 25 |
| 4 | 3 | 30 | 11 | 22 |
| 5 | 0 | 26 | 15 | 31 |
| 6 | 1 | 31 | 21 | 28 |

**Table S2 Optogenetic Activation of PrL^Pyr^ neurones delays anaesthesia induction and promotes anaesthesia emergence.**

| Group  Type  Number | mCherry | | hM3Dq | |
| --- | --- | --- | --- | --- |
|  | c-fos | neurones | c-fos | neurones |
| 1 | 1 | 29 | 18 | 30 |
| 2 | 0 | 22 | 15 | 26 |
| 3 | 1 | 29 | 18 | 29 |
| 4 | 0 | 25 | 12 | 27 |
| 5 | 2 | 28 | 10 | 23 |
| 6 | 0 | 27 | 18 | 29 |

**Table S3 Chemogenetic activation of the PrL^Pyr^-VTA neural pathway delays anaesthesia induction and promotes anaesthesia emergence.**

| Group  Type  Number | mCherry | | hM3Dq | |
| --- | --- | --- | --- | --- |
|  | c-fos | neurones | c-fos | neurones |
| 1 | 1 | 21 | 20 | 28 |
| 2 | 0 | 25 | 14 | 26 |
| 3 | 1 | 29 | 19 | 27 |
| 4 | 0 | 28 | 15 | 27 |
| 5 | 2 | 20 | 13 | 23 |
| 6 | 0 | 24 | 18 | 24 |

**Table S4 Optogenetic activation of the PrL^Pyr^-VTA neural pathway delays anaesthesia induction and promotes anaesthesia emergence.**

| Group Type  Number | mCherry | | hM3Dq | |
| --- | --- | --- | --- | --- |
|  | c-fos | neurones | c-fos | neurones |
| 1 | 0 | 28 | 11 | 33 |
| 2 | 1 | 34 | 8 | 29 |
| 3 | 1 | 32 | 9 | 28 |
| 4 | 0 | 28 | 10 | 25 |
| 5 | 0 | 25 | 11 | 25 |
| 6 | 0 | 24 | 7 | 33 |
